# Radiobiological and dosimetric assessment of DNA-intercalated ^99m^Tc-complexes bearing acridine orange derivatives

**DOI:** 10.1186/s13550-020-00663-9

**Published:** 2020-07-13

**Authors:** Ana Belchior, Salvatore Di Maria, Célia Fernandes, Pedro Vaz, António Paulo, Paula Raposinho

**Affiliations:** Centro de Ciências e Tecnologias Nucleares, Instituto Superior Técnico, Universidade de Lisboa, Estrada Nacional 10 (km 139,7), 2695-066 Bobadela LRS, Portugal

**Keywords:** Targeted radiotherapy, Radiopharmaceuticals, DNA intercalators, Auger emitters, Technetium-99m

## Abstract

**Background:**

Recently, a new family of ^99m^Tc(I)-tricarbonyl complexes bearing an acridine orange (AO) DNA targeting unit and different linkers between the Auger emitter (^99m^Tc) and the AO moiety was evaluated for Auger therapy. Among them, ^99m^Tc-C_3_ places the corresponding radionuclide at a shortest distance to DNA and produces important double strand breaks (DSB) yields in plasmid DNA providing the first evidence that ^99m^Tc can efficiently induce DNA damage when well positioned to the double helix. Here in, we have extended the studies to human prostate cancer PC3 cells using the ^99m^Tc-C_3_ and ^99m^Tc-C_5_ complexes, aiming to assess how the distance to DNA influences the radiation-induced biological effects in this tumoral cell line, namely, in which concerns early and late damage effects.

**Results:**

Our results highlight the limited biological effectiveness of Auger electrons, as short path length radiation, with increasing distances to DNA. The evaluation of the radiation-induced biological effects was complemented with a comparative microdosimetric study based on intracellular dose values. The comparative study, between MIRD and Monte Carlo (MC) methods used to assess the cellular doses, revealed that efforts should be made in order to standardize the bioeffects modeling for DNA-incorporated Auger electron emitters.

**Conclusions:**

^99m^Tc might not be the ideal radionuclide for Auger therapy but can be useful to validate the design of new classes of Auger-electron emitting radioconjugates. In this context, our results highlight the crucial importance of the distance of Auger electron emitters to the target DNA and encourage the development of strategies for the fine tuning of the distance to DNA for other medical radionuclides (e.g., ^111^In or ^161^Tb) in order to enhance their radiotherapeutic effects within the Auger therapy of cancer.

## Introduction

In the last few years, the development of internal radionuclide therapy largely contributed to the progress in the overall control of cancer and offered new possibilities for more personalized and precise treatment of malignant neoplastic diseases.

Radiopharmaceuticals used in radionuclide therapy contain therapeutic radionuclides that in some cases also emit gamma-radiation or positrons, being useful for single-photon emission computerized tomography (SPECT) or positron emission tomography (PET) imaging [1–3]. Therefore, some of those radionuclides present theranostic features allowing cancer therapy and the non-invasive monitorization of the therapeutic outcome.

Some SPECT radionuclides are also Auger electron emitters (e.g., ^99m^Tc, ^123^I, ^67^Ga, or ^111^In) and might be very interesting for selective and targeted radiotherapy due to the short penetration (< 0.1 μm) of Auger electrons in biological tissues together with their ability to induce biological effects if emitted inside the cell nucleus [4–7]. Among these radionuclides, ^99m^Tc (T_1/2_ = 6.02 h) is the most used in SPECT imaging. ^99m^Tc might not be the ideal radionuclide for Auger therapy due to its relatively low yield of Auger electrons emission (4 e- per decay) with a major portion of its decay energy being carried by photons. However, ^99m^Tc is obtained in situ at economical prices through the ^99^Mo/^99m^Tc generator and can be seen as a readily available “model” radionuclide for Auger therapy, useful to validate the design of new classes of Auger-electron emitting radioconjugates.

The biological effects induced by Auger-electron emitting radioconjugates are amplified by the emission of a cascade of Auger electrons per decay, producing clusters of DNA DSBs leading to hardly repairable and severe DNA damage in the targeted tumor cells. Neighboring healthy cells, not accumulating the radionuclides, do not suffer these effects due to the short range of Auger electrons. These features make Auger-emitting radionuclides best suited for the eradication of disseminated cancer metastases, with minimization of deleterious effect to non-target surrounding tissues.

The design of Auger-emitting radioconjugates able to selectively enter a cancer cell and reach the nucleus, being close to the DNA in order to induce effective therapeutic effects, still remains a great challenge [4–8]. To tackle this goal, several groups have shown that the use of radiolabeled DNA intercalators, able to intercalate between the DNA base pairs, is a promising approach to deliver Auger electrons to short (sub)nanomolar distances to DNA and induce lethal DNA damage [9–15].

In the past few years, we have explored this approach based on ^99m^Tc(I) tricarbonyl complexes anchored by pyrazolyl-diamine chelators and carrying AO derivatives as DNA intercalators [16–19]. In particular, we have assessed the influence of the ^99m^Tc-distance to DNA in the radiation-induced biological effects promoted by this family of ^99m^Tc(I) tricarbonyl complexes [19]. In this detailed study, the radiation-induced damage in plasmid DNA was comparatively assessed for three ^99m^Tc(I) complexes displaying alkyl linkers of different lengths (propyl (^99m^Tc-C_3_), pentyl (^99m^Tc-C_5_), and octyl (^99m^Tc-C_8_)) to attach the AO group to the pyrazolyl ring of the bifunctional chelators and for three structurally related ^125^I-labeled AO derivatives (^125^I-C_3_, ^125^I-C_5_, and ^125^I-C_8_). We verified that the plasmid DNA damage induced by the different ^99m^Tc complexes (^99m^Tc-C_3_, ^99m^Tc-C_5_, and ^99m^Tc-C_8_) was strongly dependent on the distance of the radiometal to the DNA, which was estimated based on molecular docking studies performed for the DNA-intercalated complexes. Furthermore, we demonstrated that ^99m^Tc-C_3_ and ^125^I-C_5_ place the corresponding radionuclide at similar distances to DNA and produce comparable DSB yields in plasmid DNA. It is also worthwhile to notice that we have previously shown that an AO-containing ^99m^Tc(I) complex, structurally related with ^99m^Tc-C_3_, ^99m^Tc-C_5_, and ^99m^Tc-C_8_ but carrying a bombesin (BBN) sequence, presented remarkably high cellular internalization and nuclear uptake in PC3 cells [18]. The presence of the BBN peptide provided specificity towards the gastrin releasing peptide receptor (GRPR) overexpressed in prostate cancer cells and did not compromise an extensive nuclear internalization. This was the first example of a ^99m^Tc-bioconjugate combining specific cell targeting with a pronounced nuclear internalization, showing that multifunctional radiocomplexes can transport an Auger-emitting radiometal, in a cell-specific way, to the nucleus of tumoral cells [18–20].

**Fig. 1:**
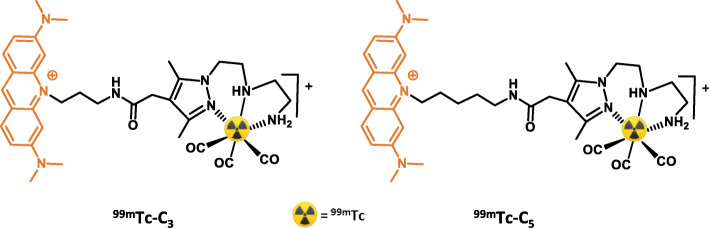
Schematic drawing of the ^99m^Tc-labeled AO derivatives studied

Based on these encouraging achievements, we have pursued with the evaluation of the radiation-induced biological effects in human prostate PC3 cancer cells for the DNA-targeted complexes ^99m^Tc-C_3_ and ^99m^Tc-C_5_ (Fig. 1). Our main goal was to evaluate the influence of the ^99m^Tc-distance to DNA on the radiobiological effects induced at the cellular level by ^99m^Tc, namely, early and late DNA damage, following our previous studies for the same compounds using plasmid DNA models. As we have reported earlier, ^99m^Tc-C_3_ places the radiometal at a shorter distance to the DNA helical axis when compared to ^99m^Tc-C_5_ (10.80 vs 12.92 Å), according to molecular modeling simulations of the DNA-intercalated complexes. The distance of Auger-electron emitting radionuclides to the DNA is a crucial issue in the design of radiopharmaceuticals for Auger therapy, together with its subcellular localization [21]. However, this issue has been rarely addressed using cellular models of cancer, as attempted in this paper. Towards this goal, we report herein on the evaluation of the cellular uptake and subcellular localization of ^99m^Tc-C_3_ and ^99m^Tc-C_5_ in human prostate cancer PC3 cells and on their radiobiological effects in the same cell line as assessed by the γ-H2AX, micronuclei, and clonogenic assays. Dosimetry studies performed at microscale will be also presented and discussed in order to rationalize the observed radiobiological effects, to evaluate the Relative Biological Effectiveness (RBE) and to suggest some mechanistic model that better could fit with the trend of experimental data.

## Methods

### Radiochemical synthesis of ^99m^Tc-C_3_ and ^99m^Tc-C_5_

The synthesis of ^99m^Tc-C_3_ and ^99m^Tc-C_5_ has been done as we have previously described [19] and as detailed in the supplementary information (SI).

### Cell studies

PC3 human prostate cancer cells (ECACC, England, UK) were grown in DMEM containing GlutaMax supplemented with 10% heat-inactivated fetal bovine serum and 1% penicillin/streptomycin antibiotic solution (all from Gibco-Invitrogen), in a humidified atmosphere of 95% air and 5% CO_2_ at 37 °C (Heraeus, Germany).

### Internalization and cellular uptake

PC3 cells were seeded at a density of 50,000 cells per well in a 24 well-plate and allowed to attach overnight. The cells were incubated with different activities (7.4 kBq–7.4 MBq) of HPLC purified ^99m^Tc-C_3_ and ^99m^Tc-C_5_ in 0.5 mL of culture medium, for 24 h at 37 °C. Incubation was terminated by washing the cells with ice-cold assay medium. Cell surface-bound radiocompound was removed by two steps of acid wash (50 mM glycine, HCl/100 mM NaCl, pH 2.8) at room temperature for 4 min. The pH was neutralized with cold PBS with 0.2% BSA, and subsequently the cells were lysed with 1 M NaOH for 10 min at 37 °C to determine the internalized radiocompound. The activity in both cell surface-bound and internalized fractions was measured.

### Nuclear uptake

PC3 cells were seeded at a density of 50,000 cells per well in a 24 well-plate and allowed to attach overnight. The cells were incubated with different activities (0.185–7.4 MBq) of HPLC purified ^99m^Tc**-**C_3_ and ^99m^Tc-C_5_ in 0.5 mL of culture medium, for 24 h at 37 °C. Incubation was terminated by washing the cells with ice-cold PBS. Cells were detached from the plates using trypsin/EDTA solution (Gibco-Invitrogen). The unbound radioactive compound was removed by centrifugation of the cell suspension, followed by washing the cellular pellet with ice-cold PBS with 0.2% BSA. The activity of cellular pellet was measured, using a gamma counter, to quantify the total cellular uptake of the radiocompound. The pellet was then resuspended in 2 mL of ice-cold cell lysis buffer (10 mM Tris, 1.5 mM MgCl_2_, 140 mM NaCl) containing 0.1% of IGEPAL-ca 630 (Sigma) and incubated on ice for 10 min to disrupt the cell membrane. After the lysis, the suspension was centrifuged at 1300*g* for 2 min at 4 °C; the supernatant (cytoplasm) was separated from the pellet (nuclei), and the activity in both fractions measured. The nuclear uptake was expressed in percentage of internalized activity.

### γ-H2AX assay and foci analysis

PC3 cells were seeded at a density of 10,000 cells per well in an eight-well chamber slide and allowed to attach overnight. Cells were incubated with several activities (0.37, 0.74, 1.85, and 3.7 MBq) of HPLC purified ^99m^Tc-C_3_ and ^99m^Tc-C_5_ in 0.3 mL of culture medium, for 24 h at 37 °C. [^99m^TcO_4_]^−^ was used as a negative control, at the same activities, since it does not efficiently internalize into cells [13] and consequently should not target the DNA to induce radiotoxic effects. PC3 cells were washed three times with PBS and fixed with 4% formaldehyde in PBS for 15 min. After washing with PBS, cells were permeabilized with Triton X-100 (0.5%) at room temperature for 5 min followed by two washing steps with 1% BSA in PBS. Then, cells were incubated with an anti-γ-H2AX primary antibody (mouse anti-γ-H2AX (ser139), Stressgen) at 2 μg/mL for 1 h. After being washed twice with 1% BSA in PBS, cells were incubated with a Texas Red-X-conjugated anti-mouse secondary antibody at 1 mg/mL for 1 h, followed by three washing steps with PBS. Cells were finally mounted in anti-fade mounting media with DAPI (Vectashield H-1200, Vector Laboratories).

Cells were analyzed at under × 64 magnification. Several high-quality 2D images were randomly collected in each slide and analyzed using the pipeline Speckle Count from the freeware CellProfiler [22]. At least 200 nuclei were analyzed per experiment per dose. Statistical analysis was performed with the Origin 7.5 software.

### Micronuclei assay

PC3 cells were seeded at a density of 50,000 cells per well in a 24 well-plate and allowed to attach overnight. Cells were incubated with several activities (0, 0.37, 0.74, 1.85, 3.7, and 7.4 MBq) of HPLC purified ^99m^Tc-C_3_ and ^99m^Tc-C_5_ in 0.5 mL of culture medium, for 24 h at 37 °C. The cytokinesis-block micronucleus (CBMN) assay allows better precision because the data obtained are not confounded by altered cell division kinetics caused by cytotoxicity of agents tested or sub-optimal cell culture conditions [23]. Cytochalasin B (Sigma-Aldrich) in a final concentration of 2 μg/mL was added to the culture medium 44 h after initial incubation to inhibit cytokinesis, allowing cells to be binucleated. After 24 h of incubation with cytochalasin B (time needed to have the majority of cells binucleated), cells were harvested by centrifugation and submitted to a mild hypotonic shock (0.075 mol/L KCl solution) to enlarge the cellular cytoplasm. The cells were then smeared onto clean glass slides, allowed to dry, fixed with methanol to glacial acetic acid (3:1), and finally stained with 4% (W/V) Giemsa (Merck; Darmstadt, Germany).

### Clonogenic assay

PC3 cells were seeded at a density of 50,000 cells per well in a 24 well-plate and allowed to attach overnight. Cells were incubated with several activities (0, 0.37, 0.74, 1.85, 3.7, and 7.4 MBq) of ^99m^Tc-C_3_ and ^99m^Tc-C_5_ in 0.5 mL of culture medium, for 24 h at 37 °C. Immediately, after incubation, cells were seeded out in appropriate dilution to form colonies, in 2 weeks, with at least 50 cells. Colonies were fixed with methanol to glacial acetic acid (3:1) and stained with Giemsa (4%).

The Plating Efficiency (PE), ratio of the number of colonies to the number of cells seeded, and the Survival Fraction (SF), number of colonies that arise after treatment of cells, expressed in terms of PE, were obtained following the methodology described in [24], where:

Eq. 1 $$ \mathrm{PE}=\frac{\mathrm{number}\ \mathrm{of}\ \mathrm{colonies}\ \mathrm{formed}\ }{\mathrm{number}\ \mathrm{of}\ \mathrm{cells}\ \mathrm{seeded}}\ \mathrm{x}\ 100\% $$

Eq. 2 $$ \mathrm{SF}=\frac{\mathrm{number}\ \mathrm{of}\ \mathrm{colonies}\ \mathrm{formed}\ \mathrm{after}\ \mathrm{treatment}}{\mathrm{number}\ \mathrm{of}\ \mathrm{cells}\ \mathrm{seeded}\ \mathrm{x}\ \mathrm{PE}} $$

### Cellular dose assessment

In order to have an estimation of the absorbed dose involved in the cellular experiments and to correlate the cell damage with the dose delivered in the two main cell structures (i.e., nucleus and cytoplasm), two methodologies for dose estimation were used. In these two methodologies, the experimental conditions were modeled with the MIRD method [25] and MCNP6 MC simulations [26], respectively. For dose calculations, the irradiation from the cell culture medium and from the cell surface-bound activity was not considered. The focus of our work was to check the influence of the distance to DNA on the doses and radiobiological effects for DNA-intercalated ^99m^Tc-complexes, mainly those associated with the emitted Auger and IC electrons. The extremely short range of Auger electrons led us to consider that the decays occurring outside the cells and at their surface should have a negligible contribution for the absorbed doses in the cell nucleus.

Aiming at performing the cellular dosimetric assessment, the cellular internalization and nuclear internalization data were used (Figs. 3 and 5). Using the final cellular activity (at a time *t* = 24 h) the starting activity *A*_0_ was calculated through the exponential decay law [27]:

Eq. 3 $$ A\ (t)={A}_0\exp \left(\raisebox{1ex}{$-0.693\ t$}\!\left/ \!\raisebox{-1ex}{${T}_p$}\right.\right) $$

where in this case *T*p is the physical half-life of ^99m^Tc (6.02 h). Knowing *A*_0_, it is possible to calculate the time-integrated activity for a given ∆*t* by integrating Eq. 3, which gives Eq. 4:

Eq. 4 $$ \tilde{\mathrm{A}}=1.44\;{T}_p\;{A}_0\left(1-\exp \left(\raisebox{1ex}{$-0.693\;\Delta t$}\!\left/ \!\raisebox{-1ex}{${T}_p$}\right.\right)\right) $$

In order to calculate the time-integrated activity in the nucleus and in the cytoplasm, the data of the internalized activity in cpm, obtained in the cellular internalization assays, were used. The conversion of cpm into becquerel was done based on the efficiency (54%) of the gamma counter used to measure the ^99m^Tc activity. Then, in order to discriminate among dose in the nucleus and in the cytoplasm, the nuclear internalization data (in percentage) were used.

### MIRD calculations

The MIRD committee of the American Nuclear Society of Nuclear Medicine is often used in order to perform dosimetry calculations at subcellular level, by using semi-analytical methods (based on the continuous slowing-down approximation) to calculate the fraction of energy released from the source that is absorbed in the target zone (S-value) [25]. The radii of cell nucleus and cytoplasm considered in this study are respectively of 2 μm and 4 μm, corresponding to the average values obtained from fluorescence microscopy images of PC3 cells, as we have described previously [28].

The source and target scenarios considered, with the respective MIRD S-value for ^99m^Tc electron spectra emission, are reported in Table 1. The ICRP-107 Auger and IC (in this study the β contribution was not taken into account given the very low radiation yield) spectra were used for this work [29]. In both source radiation scenarios (source in the nucleus and in the cytoplasm), the emitting source is supposed to homogeneously emits through all the volumes.

**Table 1:** MIRD S-values for ^99m^Tc radionuclide [41]

*S*-values (Gy/Bq□s)			
S (N ← N)	S (Cy ← N)	S (N ← Cy)	S (Cy ← Cy)
1.19E−2	1.82E−4	1.82E−4	1.74E−3

### MC simulations

**Fig. 2:**
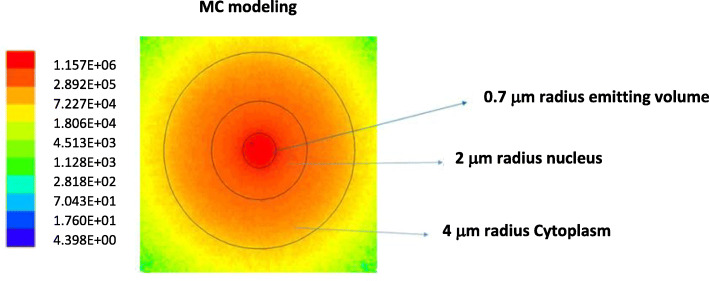
Absorbed dose distribution (in a.u.) for the spherical source emitting at the center of the nucleus. Cell dimensions (nucleus + cytoplasm) considered for MCNP6 MC simulations are also indicated

The state-of-art MC simulation program MCNP6 was used in order to calculate the absorbed doses in the volumes of interest. In MCNP6, a new single-event treatment coupled with the ENDF/B VI.8 database was developed for electron transport. This new method allows a direct sampling of microscopic data distributions and consequently an accurate low-energy transport from 1 keV down to 10 eV. ENDF/B VI.8 database contains cross sections for the atomic excitation, electron elastic scattering, subshell electro-ionization, and bremsstrahlung [30]. Further discussions about models used to obtain ENDF/B-VI-8 data library can be found in reference [31]. Single-event mode is a relatively new feature of MCNP6 that was tested and validated in several works present in literature [28, 32, 33]. A validation of the MC model was made through MC S-values calculations (for several radionuclide spectra emission), and results showed agreement with MIRD values within 3% error [28]. Figure 2 shows the geometry setup chosen for MC simulations. For the source emitting in the nucleus, an emitting spherical volume of 0.7 μm radius was modeled at the center of the cell with a nucleus of 2 μm radius and a cytoplasm of 4 μm radius. This type of setup, contrary to the homogenous source distribution in the MIRD geometry for the nucleus, was chosen in order to model a more realistic radionuclide distribution in this organelle. Instead, for the cytoplasm, the source was supposed to emit homogeneously through all the volume, as in the MIRD geometry. We would like to remark that the two dosimetric configurations considered (MIRD and MC) in this study are quite different, since in the MIRD case the source emit homogeneously in all the volume considered (nucleus or cytoplasm), whereas in the MC model the emitting source is a sphere of 0.7 μm radius (corresponding to the condensed section of chromosome) [34]. The two different configurations were considered in order to show the variability in dose estimations among two different internalization scenarios.

### RBE assessment

The RBE is a concept that determines the ratio of absorbed doses, to attain a given biological response [35]. RBE values were calculated by comparing the physical dose (D^60^Co/D^99m^Tc-C_3_/C_5_) that results in 50% survival (RBE_0.5_) and survival (SF^60^Co/SF^99m^Tc-C_3_/C_5_) for a 2 Gy dose (RBE2Gy).

Eq. 5 $$ {\mathrm{RBE}}_{0.5}=\frac{D_{60 Co}}{D_{99 mTc-C3/C5}} $$

Eq. 6 $$ {\mathrm{RBE}}_{2\mathrm{Gy}}=\frac{SF_{60 Co}}{SF_{99 mTc-C3/C5}} $$

It must be referred that for ^60^Co and ^99m^Tc-C_3_/C_5_ the modeling of the SF vs absorbed doses was made using the linear model described in [35] and detailed in SI.

## Results

### Radiosynthesis of ^99m^Tc-C_3_ and ^99m^Tc-C_5_

The synthesis of the ^99m^Tc(I) tricarbonyl complexes (^99m^Tc-C_3_ and ^99m^Tc-C_5_) was accomplished as previously described [19] by ligand-exchange reaction of *fac*-[^99m^Tc(H_2_O)_3_(CO)_3_]^+^ with the appropriate ligand, in aqueous solution at 100 °C, for 30 min and at pH = 7. Both ^99m^Tc-complexes were obtained in almost quantitative yield (> 95%) and purified by RP-HPLC in order to remove the excess of the respective chelators. After HPLC purification, ^99m^Tc-C_3_ and ^99m^Tc-C_5_ were obtained with a radiochemical purity > 99%.

### Cellular and nuclear uptake

**Fig. 3:**
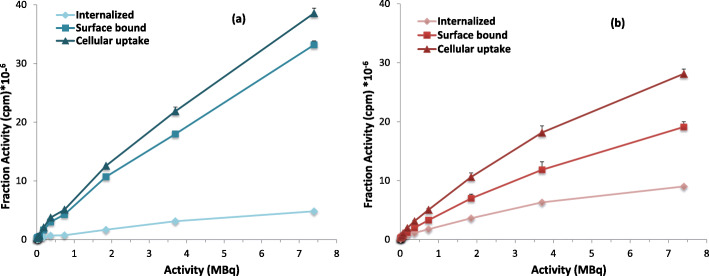
Activity-dependent cellular uptake of ^99m^Tc-C_3_ (**a**) and ^99m^Tc-C_5_ (**b**) in PC3 cells, after 24 h incubation at 37 °C. Cells were incubated with 7.4 kBq–7.4 MBq of HPLC purified radiocomplexes. Internalization, surface-bound, and cellular uptake were expressed as the activity (cpm) measured in each fraction

**Fig. 4:**
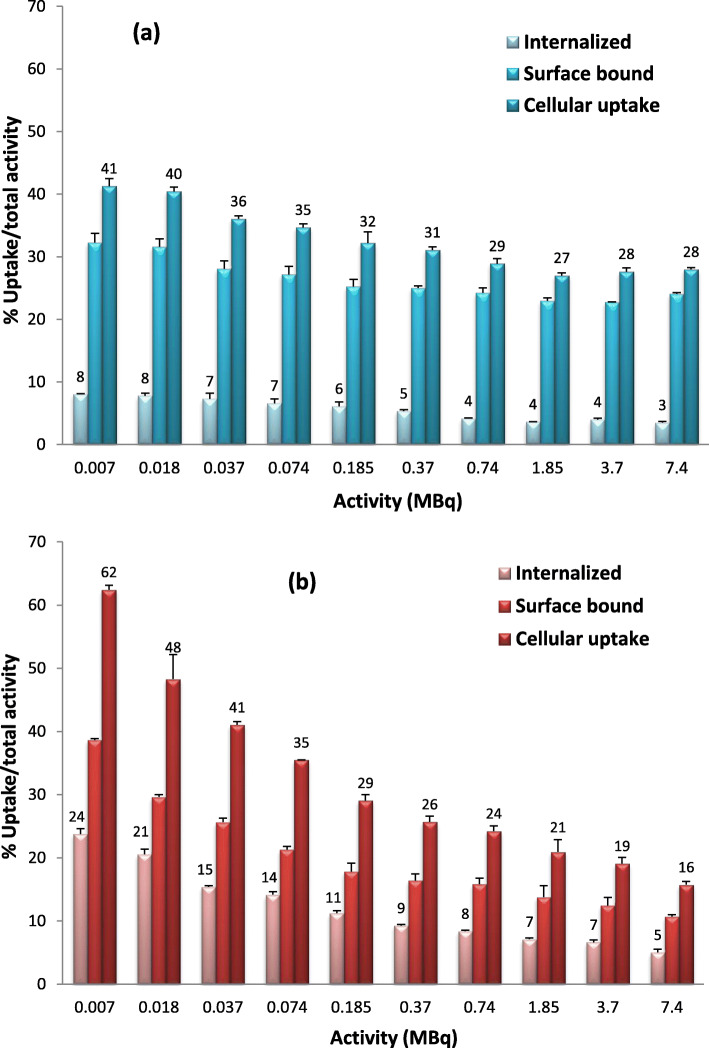
Activity-dependent cellular uptake of ^99m^Tc-C_3_ (**a**) and ^99m^Tc-C_5_ (**b**) in PC3 cells, after 24 h incubation at 37 °C. Cells were incubated with 7.4 kBq–7.4 MBq of HPLC purified radiocomplexes. Internalization, surface-bound, and cellular uptake were expressed as a percentage of total activity applied to the cells

In order to investigate the ability of ^99m^Tc-C_3_ and ^99m^Tc-C_5_ to cross the cellular and nuclear membranes, to reach the DNA target, their subcellular localization and distribution was evaluated by quantitative gamma-counting measurements, using different amounts of radioactivity. The surface-bound and internalized activities measured in cpm are represented in Fig. 3, altogether with the total cell-associated activity (cellular uptake). The internalization, surface-bound activity, and cellular uptake of ^99m^Tc-C_3_ and ^99m^Tc-C_5_ were also determined as a percentage of the applied activity (Fig. 4).

Both ^99m^Tc**-**complexes exhibited a high capacity to be associated with the PC3 cells, in a manner that is directly dependent on the applied activity (Fig. 3). As can be seen in Fig. 4, when expressed as a percentage of applied activity, the cell-association capacity (cellular uptake) of both complexes exhibited a maximum value for the lowest activities, being observed a decreasing uptake (%) with increasing applied activities. A similar trend was observed for the percentages of surface-bound and internalized activities. For an applied activity of 7.4 kBq, 41% of ^99m^Tc-C_3_ and 62% of ^99m^Tc-C_5_ were cell-associated, and 8% and 24% were able to internalize in PC3 cells, respectively, which indicates an important membrane-bound fraction. Internalized ^99m^Tc-C_5_ and ^99m^Tc-C_3_ decreased from 24 to 8% (for an applied activity of 7.4 kBq) to internalization values of 5% and 3%, respectively (for an applied activity of 7.4 MBq). For the range of tested activities (7.4 kBq–7.4 MBq), ^99m^Tc-C_5_ typically shows a 2–3 times higher capacity to internalize in PC3 cells than ^99m^Tc-C_3_, as can be seen in Figs. 3 and 4. The mechanism of cellular uptake of ^99m^Tc-C_3_ and ^99m^Tc-C_5_ was not studied in detail. However, it is not expectable that the complexes enter the cells through a specific mechanism of uptake that might justify the observed differences. Most probably, the complexes are able to enter the cells by passive diffusion as we have found for other cationic ^99m^Tc(I) complexes [36]. The measurement of the lipophilicity of both ^99m^Tc complexes has shown that ^99m^Tc-C_5_ (log *P* = 1.64 ± 0.07) is more lipophilic than ^99m^Tc-C_3_ (log *P* = − 0.044 ± 0.01) (see in the SI). This is due the presence of a pentylenic linker instead of a propylenic one and could eventually justify the highest internalization of ^99m^Tc-C_5_.

**Fig. 5:**
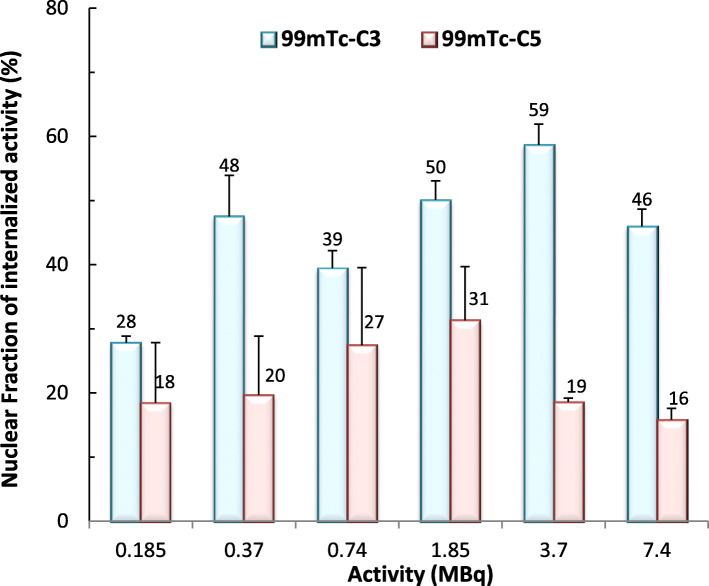
Activity-dependent nuclear uptake of ^99m^Tc-C_3_ (blue) and ^99m^Tc-C_5_ (red) in PC3 cells, after 24 h incubation at 37 °C. Cells were incubated with 0.185–7.4 MBq of HPLC purified radiocomplexes. Data expressed as a percentage of internalized activity

Nuclear uptake was expressed as a percentage of internalized activity and represented in Fig. 5. For both complexes, the nuclear fraction of the internalized activity is not strongly influenced by the amount of applied activity. After 24 h of incubation, the fraction of internalized ^99m^Tc-C_3_ that reached the nucleus (mean value 45%) was about 2 times higher than the one for ^99m^Tc-C_5_ (mean value 22%). ^99m^Tc-C_5_ internalized in PC3 cells in a higher extent than ^99m^Tc-C_3_ but, once inside the cell, the later entered more easily and/or accumulated more in the nucleus than ^99m^Tc-C_5_. Overall, the absolute activity accumulated in the nucleus is relatively similar for both compounds. For example, the activity accumulating in the nucleus was ca. 1.2 × 10^5^ and 1.4 × 10^5^ cpm for ^99m^Tc-C_3_ and ^99m^Tc-C_5_, respectively, after 24 h incubation of the PC3 cells with 0.185 MBq of each radiocomplex.

### Early DNA damage (γ-H2AX assay)

**Fig. 6:**
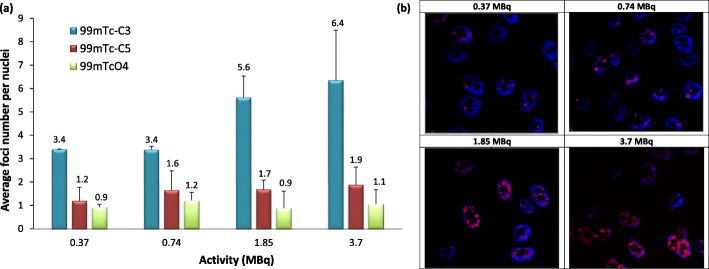
Activity-dependent DNA damage induced by ^99m^Tc-C_3_ and ^99m^Tc-C_5_ in PC3 cells_**,**_ after 24 h incubation at 37 °C. Cells were incubated with 0, 0.37, 0.74, 1.85, and 3.7 MBq of HPLC purified radiocomplexes. **a** Average number of foci per nuclei in PC3 cells exposed to ^99m^Tc-C_3_, ^99m^Tc-C_5_, and [^99m^TcO_4_]^−^. The results were normalized to control samples (i.e., non-irradiated cells). The data correspond to means ± standard deviations of 3 replicates. **b** Fluorescence images of PC3 cells exposed to 0.37–3.7 MBq of ^99m^Tc-C_3_. Cells were immunostained for γ-H2AX. DAPI was used to visualize the nuclei

The formation of γH2AX foci is the early event that follows the induction of DSBs and results from the phosphorylation of the X isoform of histone H2A at serine-139 by phosphoinositide 3-kinases [37]. Hence, the ability of ^99m^Tc-C_3_ and ^99m^Tc-C_5_ to induce DSBs in vivo, in PC3 cells, was assessed by foci analysis. [^99m^TcO_4_]^−^ was used as a negative control, at the same activities, since it does not efficiently internalize into cells [13] and consequently should not target the DNA to induce radiotoxic effects. Immediately after incubation, the cells were treated for foci quantification. For each activity, the average number of foci was calculated from the distribution of foci number per cell and normalized to control samples (Fig. 6). For the tested activities, the average number of foci per cell varied in the following ranges: (i) 2.58 ± 1.32–14.49 ± 0.91 for ^99m^Tc-C_3_; (ii) 2.72 ± 0.85–5.13 ± 0.75 for ^99m^Tc-C_5_; and (iii) 0.93 ± 0.11–2.12 ± 0.85 for [^99m^TcO_4_]^−^.

The exposure of cells to increasing activities of ^99m^Tc-C_3_ resulted in a significant increase (*p* < 0.05) in the average number of γ-H2AX foci when compared with untreated control cells. Such increase was directly dependent on the activity applied, reaching a maximum number of foci for 7.4 MBq of ^99m^Tc-C_3._ Contrarily, the number of γ-H2AX foci induced by the exposition to ^99m^Tc-C_5_ did not significantly increase for the highest applied activities. As mentioned above, ^99m^Tc-C_3_ has a higher rate of nuclear internalization than ^99m^Tc-C_5_. However, the overall accumulation of activity in the nucleus is rather similar for both complexes because ^99m^Tc-C_5_ is more prone to be internalized by the cell, giving rise to nuclear doses in the same range as discussed below. Therefore, the higher number of DNA DSBs induced by ^99m^Tc-C_3_ when compared with those induced by ^99m^Tc-C_5_ underlines the limited biological effectiveness of Auger electrons with increasing distances to DNA.

The number of γ-H2AX foci after exposure to an internalized radionuclide depends on the balance between the repaired lesions and the DSBs that are continuously formed as the radionuclide decays. Moreover, the influence of DNA repair is expected to be more important for longer incubation times [4]. These data suggest that the DNA damage induced by ^99m^Tc-C_3_, after 24 h exposition, largely exceeded the cellular capacity for its repair.

### Late DNA damage

#### Cytokinesis-block micronucleus (MN) assay

**Fig. 7:**
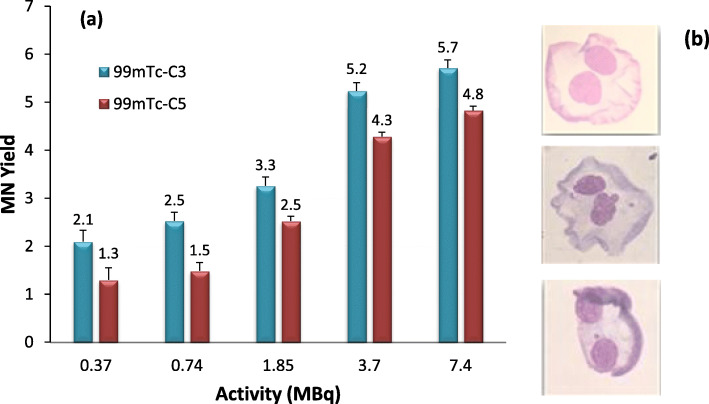
Micronucleus formation for ^99m^Tc-C_3_ and ^99m^Tc-C_5_ in PC3 cells, after 24 h incubation at 37 °C. Cells were incubated with 0.37–7.4 MBq of HPLC purified radiocomplexes. **a** Activity-dependent MN yield normalized to the control. **b** Binucleated cells without and with a MN observed at × 43-fold magnification. The data correspond to means ± standard deviations of 3 replicates

After an evaluation of the early biological effects, induced by ^99m^Tc-C_3_ and ^99m^Tc-C_5_, the late biological damage was evaluated using the MN and clonogenic assays. The MN yield was calculated using the distribution of MN number per 1000 binucleated (BN) cells and normalized to the control, as represented in Fig. 7.

Following the trend of early-induced DNA damage, the lesions observed, by micronucleus assay, for ^99m^Tc-C_3_ were more pronounced when compared with ^99m^Tc-C_5_. However, the differences were less striking than in the case of γ-H2AX foci. Micronuclei can only be expressed in cells that have completed a nuclear division. For ^99m^Tc-C_5_, the increasing number of MN with the activity suggests an accumulation of lesions, namely not repaired single-strand breaks (SSBs) that were not reflected in the early γ-H2AX quantification, which are able to induce radiobiological damage.

#### Clonogenic assay

Surviving fractions as a function of activity were determined using the clonogenic assay. After treatment, cells were plated in different dilutions and left to form colonies for 10 days.

**Fig. 8:**
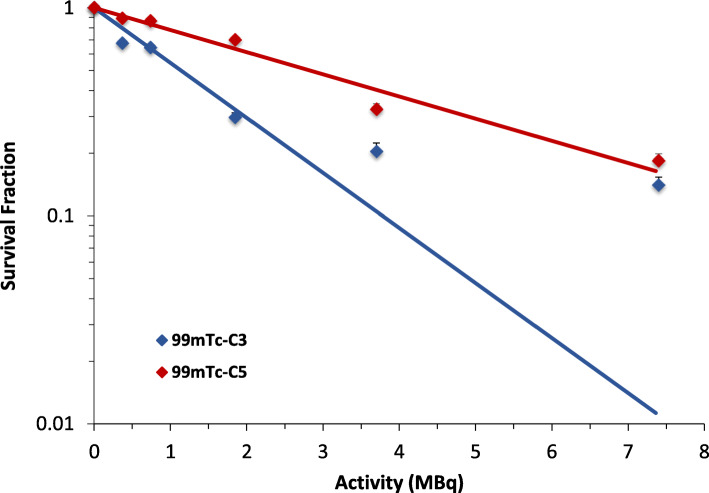
Survival Fraction from clonogenic assay experiments. PC3 cells were incubated with 0.37–7.4 MBq of HPLC purified radiocomplexes. Inhibitory potency (IC_50_) is 1.13 MBq and 2.76 MBq for ^99m^Tc-C_3_ and ^99m^Tc-C_5_, respectively. The data correspond to means ± standard deviations of 3 replicates

As can be seen in Fig. 8, an activity-dependent clonogenic survival curve was observed for both ^99m^Tc-C_3_ and ^99m^Tc-C_5_. However, for any activity used, PC3 cells exposed to ^99m^Tc-C_5_ are more viable when compared with those incubated with ^99m^Tc-C_3_. In agreement, the IC_50_ value, activity required for 50% inhibition of cell viability, was two times higher for ^99m^Tc-C_5_ than for ^99m^Tc-C_3_ (2.76 MBq versus 1.13 MBq), thereby showing the greater effectiveness of the later to induce DNA damage and inhibit cell proliferation.

We have checked the cytotoxicity of similar non-radioactive complexes, using the isostructural Re congeners, in PC3 cells and based on the MTT cell viability assay. We did not observe any cytotoxic effects after incubation of the cells with the Re complexes in concentrations ranging between 10^−11^ and 10^−6^ M, mimicking the ^99m^Tc concentrations involved in the radiotoxicity assays (data not shown). Furthermore, the ^99m^Tc-C_3_ and ^99m^Tc-C_5_ complexes were purified by HPLC to efficiently remove the unlabeled ligand. Therefore, we are confident that the results of the radiotoxicity assays are due to radiation effects and not to cytotoxicity effects inherent to the chemical nature of ^99m^Tc-C_3_ and ^99m^Tc-C_5_.

### Cell dose assessment

**Fig. 9:**
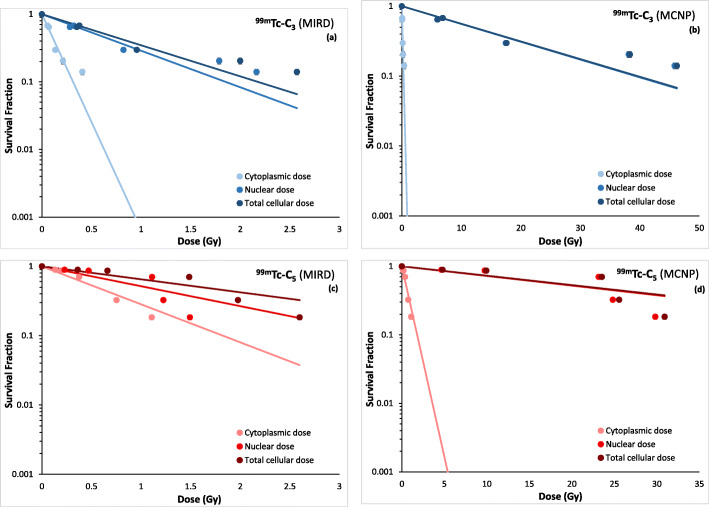
Experimental surviving fractions for ^99m^Tc-C_3_ (**a** and **b**) and ^99m^Tc-C_5_ (**c** and **d**) as a function of nuclear dose, cytoplasmic dose, and total cellular dose obtained by MIRD and MCNP6 methods, respectively

Cellular uptake and nuclear internalization data were used in order to discriminate between the dose delivered in the nucleus and cytoplasm. All the tabulated cell dose data calculated by MIRD and MCNP6 methods are reported in supplemental data [Tables SI2-SI5]. The variation of the experimental surviving fractions with the calculated doses is presented in Fig. 9, for both complexes and using the two computational methods.

For the MIRD method, the cellular, cytoplasmic, and nuclear doses (Tables SI2 and SI3) were calculated taking into account the data of activity-dependent internalization of the complexes and their nuclear uptake fractions (Figs. 3 and 5) and the MIRD S-values for ^99m^Tc. As depicted in Fig. 9, ^99m^Tc-C_3_ shows a nuclear dose distribution that is in average 85% of the total dose absorbed in the entire cell. Namely, only about 15% of the total dose is absorbed in the cytoplasm. For ^99m^Tc-C_5_, the nuclear dose is in average 65% of the total dose.

Concerning the MC calculations (Tables SI4-SI5), the data show that for both complexes, ^99m^Tc-C_3_ and ^99m^Tc-C_5_, the dose in the nucleus is more than 95% with respect to the total cell dose (nucleus + cytoplasm). In the case of MIRD dose assessment, the maximum dose value (of about 2.5 Gy) is achieved for ^99m^Tc-C_3_ with an applied activity of 7.4 MBq, whereas for MC dose assessment the maximum dose reaches the value of about 46 Gy in the cell.

### RBE assessment

As described before, RBE values, for ^99m^Tc-C_3_ and ^99m^Tc-C_5_, were calculated using Eqs. 5 and 6. It must be referred that for both ^99m^Tc-C_3_ and ^99m^Tc-C_5_, the absorbed dose values used to determine the RBE were the ones calculated using the MIRD method (Fig. 9a, c). The standard MIRD dosimetric values were preferred with respect to the MC ones in order to compare our results with the ones present in literature. Also, for these calculations, we used the total cellular dose. The reference survival curve and the irradiation conditions are described in SI.

The errors in the RBE determination were obtained by using the uncertainty of the radiobiological parameter κ in the fitted survival curves for the PC3 cells irradiated with ^60^Co, ^99m^Tc-C_3_, and ^99m^Tc-C_5_. As seen in Table 2, the RBE values are higher for ^99m^Tc-C_3_ when compared with ^99m^Tc-C_5_, both for 50% survival and for a 2 Gy dose. These results are in agreement with reports from the literature describing that internal emitters can lead to RBE values up to 4.5 [38].

**Table 2:** RBE values for ^99m^Tc-C_3_ and ^99m^Tc-C_5._ The RBE_0.5_ refers to 50% survival, and RBE_2Gy_ refers to the RBE for a 2 Gy dose

	RBE_0.5Gy_	RBE_2Gy_
^99m^Tc-C_3_	2.68 ± 0.74	3.76 ± 0.63
^99m^Tc-C_5_	1.09 ± 0.08	1.08 ± 0.17

## Discussion

Previously, we have shown that ^99m^Tc-labelled AO derivatives (^99m^Tc-C_3_ and ^99m^Tc-C_5_) can induce DSBs in plasmid DNA. However, the damage extent and the role of direct effects were strongly dependent on the linker used to attach the Auger emitting radionuclide to the AO moiety [19]. As mentioned above, molecular docking simulations revealed that ^99m^Tc-C_3_ places the ^99m^Tc atom at a shorter distance (10.80 Å) to DNA than ^99m^Tc-C_5_ (12.92 Å), enhancing the DSB yield and DNA damage by direct effects due to the emission of Auger electrons in close proximity to DNA [19]. To verify if these differences would also occur at cellular level, we have proceeded with the dosimetric and radiobiological evaluation of these two DNA-targeted ^99m^Tc-complexes in human prostate PC3 cancer cells. We have selected PC3 cells because we envisage to incorporate a targeting biomolecule (e.g., a bombesin analogue) in these complexes in order to obtain more selective radiotherapeutic effects within the Auger therapy of prostate cancer. PC3 is a very well characterized human cell line that is often used in prostate cancer research and drug development, which can be used to induce reproducible xenografts for further in vivo studies. The studies included the γH2AX, micronucleus, and clonogenic assays that provided insights into short- and long-term DNA damage and viability of the cells, upon exposure to the tested complexes.

In support of microdosimetry calculations, we have determined the subcellular localization of ^99m^Tc-C_3_ and ^99m^Tc-C_5_ based on γ-counting measurements that allow a very sensitive quantification of the radioactivity inside the cells, namely, in the nucleus which is a critical organelle in the case of Auger electron emitters [39]. ^99m^Tc-C_5_ internalizes in PC3 cells in a higher extent than ^99m^Tc-C_3_ but, once inside the cell, ^99m^Tc-C_3_ exhibited a highest nuclear fraction than ^99m^Tc-C_5_. The reasons for this difference are not clear. As we have shown previously, the congener Re complexes of ^99m^Tc-C_3_ and ^99m^Tc-C_5_ display a similar binding affinity towards duplex DNA [19]. Therefore, it is not expectable that the highest nuclear internalization of ^99m^Tc-C_3_ could be due to a possible enhanced DNA binding affinity when compared with ^99m^Tc-C_5_. Eventually, the increased ability of ^99m^Tc-C_3_ to reach the cell nuclei might be related with the use of a shortest alkyl linker (propyl vs pentanyl) to attach the AO intercalating fragment to the chelator framework. The shortest linker leads to a smaller-sized ^99m^Tc complex, which might have a more favorable size and topology to diffuse through the nuclear membrane. Nonetheless, it is important to notice that the absolute ^99m^Tc activity reaching the nucleus of PC3 cells is relatively similar for both complexes, owing to the highest cellular internalization of ^99m^Tc-C_5_.

Both ^99m^Tc-complexes are able to induce DNA-DSBs in PC3 cells. However, the average number of foci per cell is significantly higher for ^99m^Tc-C_3_ than for ^99m^Tc-C_5_ at all tested activities. Also, there is an increase of the DSBs induced by ^99m^Tc-C_3_ with increasing applied activities. On contrary, ^99m^Tc-C_5_ induces a rather similar number of DSBs for all applied activities. For longer incubation times, as those used in this work, there is a balance between the repaired foci and the ones continuously formed due to the radionuclide decays. Our results indicate that the influence of DNA repair is more evident for ^99m^Tc-C_5_. It is well established in the literature that high LET radiation, as Auger electrons, induce clusters of DNA damage that compromises DNA repair. According to our previous in silico molecular docking studies, ^99m^Tc is placed closer to the DNA axis in the case of ^99m^Tc-C_3_ when compared with ^99m^Tc-C_5_. Hence, ^99m^Tc-C_3_ is more prone to deposit the ^99m^Tc decay energy within the DNA structure and, therefore, can induce more complex lesions. This could explain the increasing number of DNA lesions leading to more important early and late cellular damage.

The dose values calculated by the MIRD and MC methods present a difference of about one order of magnitude. This difference clearly shows how sensitive is the calculation method with respect to the final dose values with a consequent effect on the surviving fraction curves as a function of nuclear, cytoplasmic, and cellular doses (see Fig. 9). Despite the different distribution of ^99m^Tc-C_3_ and ^99m^Tc-C_5_ between the main cellular compartments, the nucleus, and the cytoplasm, the energy deposition is highly localized in the nucleus for both complexes. This is due to the models considered (MIRD and MC) and reflects the physics of Auger electrons interaction in water (range of the order of few nanometers).

In support of the higher biological effectiveness of ^99m^Tc-C_3_ when compared with ^99m^Tc-C_5_, verified by the early and late DNA damage and clonogenic assays, the RBE assessment indicates RBE values significantly higher for ^99m^Tc-C_3_ than for ^99m^Tc-C_5_. These RBE values are in agreement with other studies in the literature [38] and reveal that internal emitters, in particular DNA-incorporated Auger emitters, can induce lethal lesions if placed in close proximity to the DNA helix.

However, it is worthwhile to mention that the performed calculations have some inherent limitations. Considering the uncertainties in the RBE calculations, the methods used to assess the absorbed doses for the cells irradiation with ^60^Co and ^99m^Tc were different. For ^60^Co, we used an ionization chamber to obtain the dose at the cellular monolayer, while for the ^99m^Tc-complexes the dose was calculated by the MIRD method. Another limitation is the assumption of homogenous or non-homogeneous dose distribution inside the cells. In fact, for internal irradiation of cells with radionuclides, it is not possible to fully quantify the real dose distribution inside the cells, namely, in target organelles (ex. nucleus, cytoplasm, mitochondria, among others) with different radiosensitivity.

## Conclusions

A detailed biological evaluation of the DNA-intercalated ^99m^Tc-C_3_ and ^99m^Tc-C_5_ complexes in human prostate PC3 cancer cells showed that ^99m^Tc-C_3_ leads to more pronounced radiation-induced biological effects when compared with ^99m^Tc-C_5_. These results show that there is a marked dependence of the biological effectiveness of ^99m^Tc Auger electrons on the ^99m^Tc-DNA distance, even for a relatively small increase of such distance (10.80 Å vs 12.92 Å for ^99m^Tc-C_3_ and ^99m^Tc-C_5_, respectively), as expected for short path length radiation.

Both methods (MIRD and MC) used to perform the microdosimetry study point to greater dose values in the nucleus (with respect to cytoplasm) for ^99m^Tc-C_3_ or ^99m^Tc-C_5_. Nevertheless, our comparative study revealed that efforts should be made in order to standardize the bioeffects modeling for DNA-incorporated Auger electron emitters, namely, to address the nano-scopic pattern of the emitted short-range electrons.

The highest biological effectiveness found for ^99m^Tc-C_3_ makes this complex more promising for further investigation within anticancer Auger therapy. ^99m^Tc-C_3_ is not expected to display by itself preferential tumor cell uptake relatively to normal cells. However, this complex can be endowed with specificity towards tumoral cells upon functionalization with targeting biomolecules. Their selection depends on the type of cancer we want to target (and to threat). To profit from the Auger effect is crucial that the targeting molecules undergo extensive internalization, such as somatostatin or bombesin analogues with encouraging results in Peptide Receptor Radionuclide Therapy (PRRT) using β-emitters [40]. Having this in mind, we are pursuing with the functionalization of ^99m^Tc-C_3_ with bombesin derivatives.

Our results highlight the crucial importance of the distance of Auger electron emitters to the target DNA and encourage the development of strategies for the fine tuning of the distance to DNA for other medical radionuclides (e.g., ^111^In or ^161^Tb) [6] in order to enhance their radiotherapeutic effects within the Auger therapy of cancer.

## Supplementary information

**Additional file 1: Fig. SI1.** Radiochemical synthesis of ^99m^Tc-C_3_ and ^99m^Tc-C_5_. **Fig. SI2.** Survival curve for the PC3 cell line obtained by clonogenic assay, using ^60^Co. The points represent the mean value of three independent experiences and the s.e.m. is represented by the error bars. **Table SI1.** κ parameter for ^60^Co, ^99m^Tc-C_3_ and ^99m^Tc-C^5^.** Table SI2**. Absorbed doses calculated through MIRD method for ^99m^Tc-C_3_. Table SI3. Absorbed doses calculated through MIRD method for ^99m^Tc-C5. Table SI4. Absorbed doses calculated through MCNP6 MC Simulations for ^99m^Tc-C_3_. Table SI5. Absorbed doses calculated through MCNP6 MC Simulations for ^99m^Tc-C5.

## Data Availability

All the data is reported on the manuscript and in the additional Supplementary Information file submitted.

## Abbreviations

AO: Acridine orange
DSB: Double strand breaks
MC: Monte Carlo
SPECT: Single-photon emission computerized tomography
BBN: Bombesin
PE: Plating Efficiency
SF: Survival Fraction
RBE: Relative Biological Effectiveness
