# Supplementary material for: Radiobiological and dosimetric assessment of DNA-intercalated 99mTc-complexes bearing acridine orange derivatives
Source: EJNMMI Res. 2020 Jul 13;10:79. doi: 10.1186/s13550-020-00663-9 (PMC7359215; doi:10.1186/s13550-020-00663-9)
Supplement: Supplementary file 1 — Additional file 1: Fig. SI1. Radiochemical synthesis of 99mTc-C3 and 99mTc-C5. Fig. SI2. Survival curve for the PC3 cell line obtained by clonogenic assay, using 60Co. The points represent the mean value of three independent experiences and the s.e.m. is represented by the error bars. Table SI1. κ parameter for 60Co, 99mTc-C3 and 99mTc-C5. Table SI2. Absorbed doses calculated through MIRD method for 99mTc-C3. Table SI3. Absorbed doses calculated through MIRD method for 99mTc-C5. Table SI4. Absorbed doses calculated through MCNP6 MC Simulations for 99mTc-C3. Table SI5. Absorbed doses calculated through MCNP6 MC Simulations for 99mTc-C5. [file 13550_2020_663_MOESM1_ESM.docx]

**Supplementary Information (SI)**

*Radiochemical Synthesis of ^99m^Tc-C_3_ and ^99m^Tc-C_5_:* The radiosynthesis and purification of the ^99m^Tc complexes was conducted according to the methods that we have previously described [1]. Briefly, the radioactive precursor *fac*-[^99m^Tc(CO)_3_(H_2_O)_3_]^+^ was prepared by reaction of Na[^99m^TcO_4_] (3 mL) with a mixture of sodium boranocarbonate (4.5 mg), sodium tartrate (8.5 mg), sodium carbonate (7.2 mg) and sodium tetraborate (2.9 mg) during 30 min at 100ºC. The radioactive precursor *fac*-[^99m^Tc(CO)_3_(H_2_O)_3_]^+^ (1.5 mL, pH =7) was then added to a 10^-3^ M ethanolic solution of the **L-C_3_** or **L-C_5_** ligand for 30 min at 100°C, giving the corresponding **^99m^Tc-C_3_** and **^99m^Tc-C_5_** complexes, which were purified by RP-HPLC (**^99m^Tc-C_3_**: ***t*_R_=**21.6 min; **^99m^Tc-C_5_**: ***t*_R_=**21.8 min).


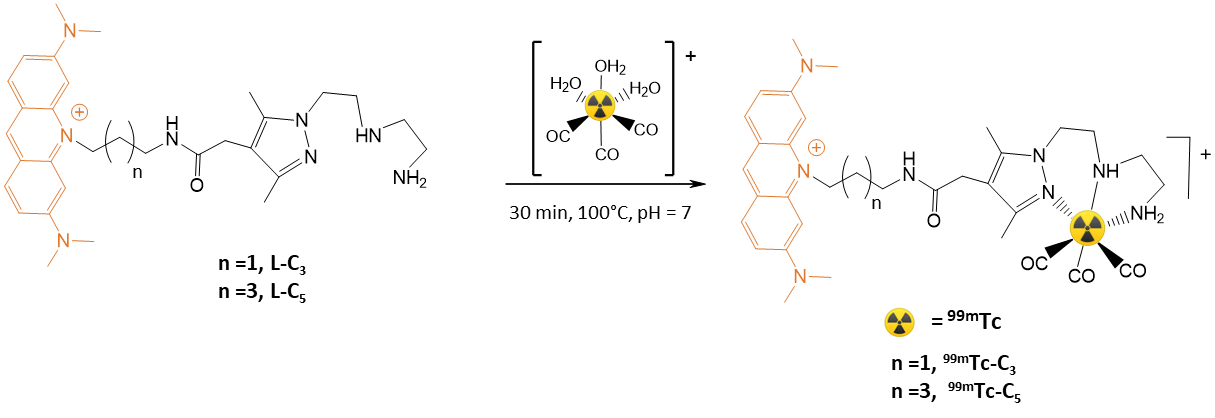


**Fig. SI1.** Radiochemical synthesis of **^99m^Tc-C_3_** and **^99m^Tc-C_5_**.

*Lipophilicity:* The lipophilicity of complexes **^99m^Tc-C_3_** and **^99m^Tc-C_5_** was assessed by the “shake-flask” method [2]. Briefly, a mixture of n-octanol (1.0 mL) and 0.1 M PBS pH = 7.4 (1.0 mL) was stirred vigorously to saturate the phases in each other. Thereafter, 50 μL of the solution of each compound was added and the mixture vortexed and centrifuged (3000 rpm, 10 min, room temperature) to allow phase separation. Aliquots of 100 μL of the octanol and PBS phases were counted in a gamma counter. The partition coefficient (Po/w) was calculated by dividing the counts in the n-octanol phase by those in the aqueous buffer, and the results expressed as Log Po/w. Log Po/w= -0.04 ± 0.01 (**^99m^Tc-C_3_**); Log Po/w = 1.64 ± 0.07 (**^99m^Tc-C_5_**).

*RBE assessment:* For the calculation of RBE values the reference radiation used was ^60^Co. The irradiations were carried out with the experimental equipment Precisa 22 (Graviner, Manufacturing Company Ltd, U.K.) [3], loaded with 4 ^60^Co sources, located at Campus Tecnológico e Nuclear (CTN), Portugal. The dose rate used was 1.15-1.18 Gy/min. The sources had an activity of 98 TBq at 1^st^ of March and 97 TBq at 1^st^ of April of 2018. The time required was calculated for the doses used in the present work: 0.5, 1, 2, 4, 6 and 10 Gy.


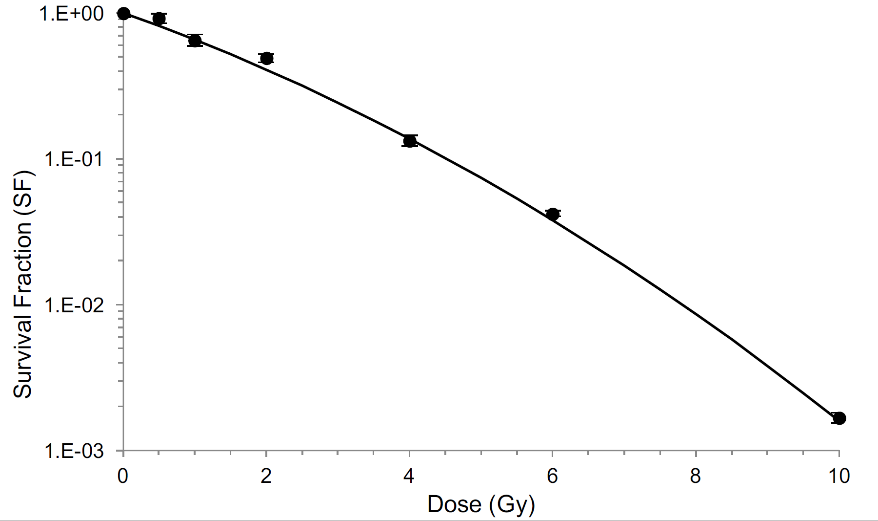


**Fig. SI2.** Survival curve for the PC3 cell line obtained by clonogenic assay, using ^60^Co. The points represent the mean value of three independent experiences and the s.e.m. is represented by the error bars.

Since a low degree of curvature was observed with the SF obtained with ^99m^Tc Auger emitter radionuclide compounds, a linear model was preferred with respect to linear quadratic one [4]. For comparison reasons, this linear model was used both for radionuclide compounds and ^60^Co irradiation configurations. Specifically, the SF was modelled according to the following equation:

$SF=e^{-\kappa D_{H}}$ (Eq. SI1)

where κ is the radiological parameter that describes the radiobiological response for DNA-incorporated Auger electron emitters and $D_{H}$ represents the absorbed dose from high-LET radiation. The κ values for ^60^Co, and **^99m^Tc-C_3_/C_5_** are depicted in Table SI1.

Table SI1. κ parameter for ^60^Co, **^99m^Tc-C_3_** and **^99m^Tc-C_5_**

|  | κ (Gy^-1^) |
| --- | --- |
| ^60^Co | 0.3952 (±0.0613) |
| **^99m^Tc-C_3_** | 1.0576 (±0.1555) |
| **^99m^Tc-C_5_** | 0.4319 (±0.0579) |

*Cellular dose assessment*

**Table SI2.** Absorbed doses calculated through MIRD method for **^99m^Tc-C_3_**.

| **Applied activity**  **(MBq)** | **Dose nucleus (Gy)** | **Dose cytoplasm (Gy)** | **Total dose (Gy)** | **Mean cell dose** |
| --- | --- | --- | --- | --- |
| 0.37 | 0.322 | 0.056 | 0.378 | 0.089 |
| 0.74 | 0.282 | 0.069 | 0.351 | 0.095 |
| 1.85 | 0.822 | 0.134 | 0.956 | 0.220 |
| 3.7 | 1.788 | 0.212 | 1.999 | 0.409 |
| 7.4 | 2.164 | 0.407 | 2.572 | 0.626 |

**Table SI3.** Absorbed doses calculated through MIRD method for **^99m^Tc-C5**.

| **Applied activity**  **(MBq)** | **Dose nucleus (Gy)** | **Dose cytoplasm (Gy)** | **Total dose (Gy)** | **Mean cell dose** |
| --- | --- | --- | --- | --- |
| 0.37 | 0.228 | 0.132 | 0.360 | 0.144 |
| 0.74 | 0.471 | 0.190 | 0.661 | 0.225 |
| 1.85 | 1.110 | 0.375 | 1.485 | 0.466 |
| 3.7 | 1.225 | 0.752 | 1.977 | 0.810 |
| 7.4 | 1.492 | 1.107 | 2.599 | 1.153 |

**Table SI4.** Absorbed doses calculated through MCNP6 MC Simulations for **^99m^Tc-C_3_**.

| **Applied activity**  **(MBq)** | **Dose nucleus (Gy)** | **Dose cytoplasm (Gy)** | **Total dose (Gy)** | **Mean cell dose** |
| --- | --- | --- | --- | --- |
| 0.37 | 6.825 | 0.054 | 6.880 | 0.900 |
| 0.74 | 5.948 | 0.067 | 6.015 | 0.802 |
| 1.85 | 17.456 | 0.128 | 17.584 | 2.294 |
| 3.7 | 38.130 | 0.199 | 38.329 | 4.940 |
| 7.4 | 45.839 | 0.392 | 46.230 | 6.072 |

**Table SI5.** Absorbed doses calculated through MCNP6 MC Simulations for **^99m^Tc-C5**.

| **Applied activity**  **(MBq)** | **Dose nucleus (Gy)** | **Dose cytoplasm (Gy)** | **Total dose (Gy)** | **Mean cell dose** |
| --- | --- | --- | --- | --- |
| 0.37 | 4.648 | 0.131 | 4.779 | 0.695 |
| 0.74 | 9.751 | 0.187 | 9.938 | 1.382 |
| 1.85 | 23.160 | 0.367 | 23.527 | 3.215 |
| 3.7 | 24.820 | 0.744 | 25.563 | 3.752 |
| 7.4 | 29.819 | 1.097 | 30.916 | 4.685 |

**References**

1. Pereira E, Quental L, Palma E et al.. Evaluation of Acridine Orange Derivatives as DNA-Targeted Radiopharmaceuticals for Auger Therapy: Influence of the Radionuclide and Distance to DNA. Sci. Rep. 2017;7:42544.
2. Troutner DE, Volkert WA, Hoffman TJ, Holmes RA. Int. J. Appl. Rad. Isot. 1984;35:467-470.
3. Belchior A, Botelho ML, Vaz P. Monte Carlo simulations and dosimetric studies of an irradiation facility. Nucl. Instrum. Methods Phys. Res. A 2007;580:70-72.
4. Hobbs R, Howell R, Song H et al. Redefining Relative Biological Effectiveness in the Context of EQDX Formalism: Implications for Alpha-Particle Emitter Therapy. Radiat. Res. 2014;181(1):90-98.
